# Quaternary Ammonium-Functionalized Chitosan as a Next-Generation Antifungal Platform: Chemistry, Mechanisms, and Therapeutic Applications

**DOI:** 10.3390/md24070249

**Published:** 2026-07-17

**Authors:** Neha Jain, Shreya Kaul, Rupali Verma, Triveni, Krishna Kant Jangde, Unnati Garg, Dinesh Kumar Mishra, Upendra Nagaich, Mahmoud H. Abu Elella

**Affiliations:** 1 Center for Pharmaceutics, Amity Institute of Pharmacy, Amity University, Noida 201313, Uttar Pradesh, India; rupaliv284@gmail.com; 2 Amity Institute of Pharmacy, Amity University, Kolkata 700135, West Bengal, India; ttiwari@kol.amity.edu; 3 Department of Pharmacy, Guru Ghasidas Vishwavidyalaya (A Central University), Bilaspur 495009, Chhattisgarh, India; kjangde10@gmail.com (K.K.J.); dineshdops@gmail.com (D.K.M.); 4 Department of Management, Amity University Online, Noida 201313, Uttar Pradesh, India; gargunnati143@gmail.com; 5 Dr. D. Y. Patil Medical College, Hospital and Research Centre, Dr. D. Y. Patil Vidyapeeth, Pimpri, Pune 411018, Maharashtra, India; upendra_nagaich@hotmail.com; 6 Chemistry Department, Faculty of Science, Cairo University, Giza 12613, Egypt

**Keywords:** quaternary ammonium chitosan, chitosan quaternization, N,N,N-trimethyl chitosan (TMC), quaternized chitosan derivatives, fungal biofilms, antifungal resistance

## Abstract

Fungal infection remains a significant therapeutic concern owing to the scarcity of drugs, resistance development, and high levels of toxicity of many conventional antifungals. In this regard, chitosan is one such natural polymer whose biocompatibility, biodegradability, and antimicrobial nature have made it the focus of scientific interest. However, the main problem lies in the polymer’s limited aqueous solubility under physiological conditions and its poor efficacy against fungi. Thus, quaternary ammonium functionalization represents a convenient approach to solving the problem by introducing permanent positive charges into the molecular structure of chitosan, thereby improving water solubility, enabling membrane interactions, and conferring broad-spectrum antifungal activity. Such derivatives can exhibit strong interactions with the negatively charged surface of fungal cells, compromising membrane integrity and inhibiting biofilm formation. Apart from their antifungal activity, such compounds are also promising in local delivery systems, which include coatings, nanoparticles, hydrogels, and wound dressings. Considering their unique chemistry, such compounds appear quite promising for the development of novel antifungal biomaterials that can be adapted to different clinical and pharmaceutical needs. Overall, quaternary ammonium-modified chitosan could be considered a promising platform for the development of next-generation antifungal agents. This review offers a comprehensive discussion regarding the synthesis, mode of action, formulation advancements, safety profile, potential applications, and future directions of quaternary ammonium-functionalized chitosan derivatives as an antifungal agent, with an emphasis on artificial intelligence contributions to this area of research.

## 1. Introduction

Fungal infections have become a serious global public health problem. Initially, these were opportunistic infections that affected only immunocompromised patients, but now, invasive fungal infections (IFIs) have been confirmed as a significant source of global morbidity and mortality. A systematic review carried out by Denning et al. in 2024 estimates that more than 6.5 million individuals are affected by IFIs annually, resulting in more than 3.8 million deaths; thus, the disease burden is comparable to that of tuberculosis and greater than that of certain other infectious diseases [1].

The global burden of IFIs is largely attributable to a few pathogenic fungal species. For instance, invasive aspergillosis is reported to account for about 2.1 million cases annually, characterized by an 85.2% case-fatality rate and associated with 1.8 million annual mortalities [1]. Invasive candidiasis due to *Candida albicans* and drug-resistant *Candida auris* is another cause, while cryptococcal meningitis causes about 180,000 deaths annually among HIV-positive patients [2]. Mucormycosis, pneumocystosis, histoplasmosis, and coccidioidomycosis are also contributing factors to the IFI burden on the global population [3].

In response to the emerging risk of pathogenic fungi, the World Health Organization (WHO) released the world’s first Fungal Priority Pathogen List, in which it classified 19 fungal species based on their public health significance and susceptibility to antifungals in 2022. These species were ranked as critical-priority pathogens based on their significant mortality and treatment difficulty. The four critical-priority pathogens include *Cryptococcus neoformans*, *C. auris*, *Aspergillus fumigatus*, and *C. albicans* [4]. The growing prevalence of fungal infections is associated with an increase in vulnerable patient populations, such as those receiving chemotherapy, organ transplantation, corticosteroid therapy, and biological agents, and people with HIV/AIDS [5]. The coronavirus disease (COVID-19) pandemic has brought attention to the problem of secondary opportunistic infections among COVID-19 patients, such as COVID-19-associated pulmonary aspergillosis and mucormycosis [6]. Moreover, climate change could pose additional threats to public health by creating new habitats for thermotolerant fungi [7].

Although the prevalence of fungal infections has become more prominent, there have been no discoveries that can significantly expand the current list of antifungals, which only consists of polyenes (amphotericin B), azoles, echinocandins, and flucytosine [8,9]. In comparison to antibacterial drugs, which are very diverse, the narrow variety of antifungal drugs is notable. Unfortunately, each of these antifungal drug classes poses notable limitations. While amphotericin B displays potent fungicidal action, its use is characterized by nephrotoxicity, infusion complications, and electrolyte imbalance [10]. Though lipid formulation offers greater tolerability, the expensive price tag limits availability in low- and middle-income nations. Azoles, despite being the most used antifungals because of oral administration, are only fungistatic and pose a risk of multiple drug–drug interactions, as well as causing hepatotoxicity and QT prolongation [11]. Echinocandins are safe antifungals, but there are no oral preparations in the group, and they have poor activity against the *Cryptococcus genus* and some filamentous fungi [9].

One serious issue in recent years has been antifungal resistance [12]. For example, azole resistance in *A. fumigatus*, caused mainly by CYP51A mutations and exposure to azoles used in agriculture, has been observed at a prevalence of 15–20% in certain regions of Europe, with rates of up to 80–90% in some environmental isolates of Southeast Asia [13,14]. Likewise, nearly 90% of *Candida auris* isolates are resistant to fluconazole, with multidrug resistance and pan-drug (polyenes, azoles and echinocandins) resistance also becoming increasingly common across the world [15]. The mechanisms behind antifungal resistance include target-site mutations, upregulation of efflux pumps, and changes in ergosterol and cell-wall biosynthetic pathways [16,17]. In addition to the problems in therapeutic intervention, late diagnosis still presents another major challenge. Laboratory confirmation for invasive fungal infections takes time, sometimes even a few days, leading to unnecessary use of antifungals in empirical treatments and eventually the development of resistance. Moreover, the means of diagnostic facilities and the availability of antifungal drugs are limited in many resource-limited settings [2,18].

In view of the constraints of traditional antifungal agents, there is increasing interest in using polymers endowed with inherent antifungal properties, where the antimicrobial activity stems from their chemical composition and not the presence of drug molecules. Such polymers have several desirable characteristics, such as a multistage mechanism of action, low probability of developing resistance, tunable physicochemical characteristics, facile chemically modifiable structures, drug delivery compatibility, and suitability for medical dressings, devices, and antifungal coatings [19,20]. There is a wide variety of such polymers with antifungal properties under investigation. Cationic polymers such as polyethyleneimine (PEI), poly (diallyl dimethylammonium chloride) (PDADMAC), and amphiphilic block copolymers can exert an antifungal effect by interacting electrostatically with negatively charged cell walls [20,21]. Similarly, poly (β-amino esters) and β-peptide polymers, namely poly (DL-diaminopropionic acid) (PDAP), have been found to possess strong antifungal activity due to their ability to disrupt membranes, penetrate cells, generate reactive oxygen species (ROS), and induce apoptosis [22]. Other types of polymers with antifungal effects include cellulose acetate fibers impregnated with essential oils and polylactic acid (PLA)-based antimicrobial nanocomposites [23]. Nonetheless, there are certain limitations associated with the use of synthetic cationic polymers, including cytotoxicity, lack of biodegradability, persistence in the environment, and other regulatory constraints that have hampered the translation of research findings into practice [22]. As a result, researchers have increasingly focused on natural biopolymers, which offer antimicrobial efficacy alongside good biocompatibility and biodegradability.

Chitosan is one of the most effective antifungal biopolymers owing to its biological, chemical, and physicochemical attributes [24]. It is a linear polyelectrolyte polysaccharide made up of β-(1→4)-linked D-glucosamine and N-acetyl-D-glucosamine residues, formed via deacetylation of chitin, which is the second most common natural biopolymer, present in the exoskeletons of crustaceans, insect cuticles, and fungal cell walls. The degree of deacetylation (DD) of chitosan, usually 70–95%, defines its fundamental physicochemical characteristics, including solubility, charge density, and biological activity. Due to its remarkable combination of biocompatibility, biodegradability, immunogenicity, mucoadhesiveness, and a broad range of biological functions (e.g., antimicrobial, antifungal, anti-inflammatory, and wound-healing activity), chitosan has been extensively studied for biomedical applications [14,24]. The antifungal property of chitosan is achieved mainly due to the electrostatic interactions between positively charged groups in the polymer (-NH_3_^+^) and negatively charged fungal membranes, causing damage to their structure, higher membrane permeability, and discharge of their intracellular contents [24,25]. Other mechanisms of action include metal chelation, inhibition of spore germination and respiration, and downregulation of various fungal genes responsible for pathogenesis, cell-wall integrity, and drug resistance [26,27].

It is important to highlight that amongst several cationic antimicrobial polymers such as poly-L-lysine (PLL), polyethyleneimine (PEI), and chitosan and its synthetic quaternary ammonium salt. Chitosan belongs to a naturally derived biodegradable, biocompatible, and poorly soluble polysaccharide, which can be functionalized with quaternary ammonium groups to selectively enhance its solubility and positive charge density to bind specifically to negatively charged fungal cell surfaces. In comparison, synthetic polymers like PEI demonstrate their antifungal activity via high cationic charge but suffer from substantial cellular toxicity, low blood compatibility, and poor biodegradability [28].

Chitosan-based nanoparticles are effective antifungal agents against clinically significant fungi, including *C. albicans*, *A. fumigates*, *Aspergillus niger*, and *Mucor circinelloides*, with potent activity being largely dependent upon the cationic charge density and surface area of the particles [29,30]. Even with all these benefits, there are several limitations associated with native chitosan. The compound’s solubility is mainly associated with acidic pH (pH < 6), and its ability to precipitate at physiological pH decreases its antimicrobial activity. In addition, the antifungal effect of native chitosan may not be sufficient for resisting some antifungal-resistant fungi. Therefore, a lot of research efforts have focused on modifying the chemical structure of chitosan to enhance its water solubility and antifungal activity [27,29,30,31]. The polymer backbone of chitosan contains three highly reactive functional groups: one amino group at the C-2 position and hydroxyl groups at the C-3 and C-6 positions. These reactive sites provide extensive opportunities for structural tailoring through chemical derivatization.

As part of the many chemical modifications that have been applied to chitosan, the quaternary ammonium modification approach has proved to be one of the most successful ways to improve its antifungal properties. The process of quaternization makes the chitosan macromolecule bear permanent positive charges by way of N-alkylation of the amino groups, thus making chitosan lose its pH dependence and acquire pH independence, as well as becoming water-soluble and highly charged [32,33]. Examples of such derivatives include N,N,N-trimethyl chitosan (TMC), hydroxypropyltrimethyl ammonium chloride chitosan (HACC), and pyridinium chitosan, among others [32,34]. The enhanced antifungal activity of quaternary ammonium chitosan (QAC) is due to various synergistic effects. The presence of a permanent positive charge increases the aqueous solubility at physiological pH values and facilitates increased electrostatic interaction with negative charges present in the fungal membrane, leading to higher membrane disruption and cell death [32,35]. Moreover, different degrees of functionalization and types of quaternary ammonium groups enable control over charge density, hydrophobicity, and affinity for membranes, facilitating a range of structure–activity optimization opportunities [36]. Significantly, many QAC derivatives display high potency against fungal biofilms that are generally highly resistant to conventional antifungal agents [26,37]. In addition to their inherent antifungal properties, QAC derivatives are extremely versatile in terms of formulation possibilities and have been used for incorporation into nanoparticles, hydrogels, films, coatings, and electrospun nanofibers designed specifically for antifungal applications. The combination of a broad spectrum of antifungal activity against yeast, mold, biofilms, and drug-resistant fungi, along with high biocompatibility and physicochemical characteristics, makes QAC derivatives extremely attractive candidates for future antifungal agents and biomaterials [36,37,38].

The success of the translation of QAC into the clinic relies on its safety and biocompatibility properties. Taking advantage of the positive safety profile of chitosan itself, many QAC derivatives exhibit low cytotoxicity and excellent hemocompatibility in concentrations where antifungal activity is seen [39]. Due to the biodegradable nature of chitosan derivatives, the risks related to the usage of traditional synthetic quaternary ammonium compounds are even lower. Nonetheless, the safety of such materials is highly dependent on structural features, since high substitution degree, charge density, and large hydrophobic alkyl groups increase cytotoxicity [40,41]. Also, ecotoxicology studies on QACs remain insufficient [42]. QACs’ classification according to regulations is determined by their intended usage in biomedical technology, where they can be categorized as a pharmaceutical excipient, an active pharmaceutical ingredient, a drug delivery system, or even a medical device coating [38].

To establish the uniqueness of this review and clearly state how our work differs from and advances beyond the existing literature, we conducted a literature survey. Google Search, Google Scholar, Scopus, and PubMed were used to find significant results at the scientific level. The following keywords were searched in the literature review: “quaternary ammonium chitosan”, “drug delivery”, “antimicrobial”, “antifungal,” and so on. The inclusion criteria were review-based articles. The search was restricted to the last five years. PubMed yielded no review articles related to this search. We got only five review articles, as compiled in Table 1.

Although it has good antifungal potential, many limitations still hamper the development of QAC as a drug delivery vehicle. Some key areas where more knowledge is required include the incomplete understanding of the mode of action of QAC, lack of adequate structure–activity relationship (SAR) studies, absence of sufficient in vivo data, and lack of comprehensive research regarding antifungal drug delivery systems, biofilm prevention, development of drug resistance, and safety [32,35,36,47]. In addition, much of the literature is highly scattered, with existing reviews focusing mainly on general antimicrobial properties of chitosan or different methods of formulation of the compound, but not on QAC-functionalized chitosan as antifungals [48,49]. Thus, the current review gives a comprehensive overview of the chemistry of QAC derivatives, synthesis approaches, SARs, mechanisms of action, preclinical results regarding antifungal activity, formulation approaches, safety, and therapeutic uses of the compounds.

## 2. Structural Modification of Chitosan and Its Impact on Antifungal Activity

Several chemical modification approaches have been explored for enhancing the functionality of chitosan. The significant versatility of chitosan largely arises from the presence of reactive amino and hydroxyl functional groups within its polymeric backbone. Because of the presence of these functional groups, chitosan can be chemically modified, producing chitosan derivatives with improved physicochemical, biological, and pharmaceutical properties (Table 2). Furthermore, it is possible to chemically modify native chitosan to improve its disadvantages related to low solubility at physiological pH, low mechanical properties, and low stability in certain biomedical/biopharmaceutical applications.

### 2.1. Quaternization Synthesis Strategies of Chitosan

Quaternization is one of the most important modifications for improving the physicochemical and biological properties of chitosan. However, primary amino groups on the native chitosan are protonated only in acidic media, limiting its solubility and antimicrobial activity at neutral pH values. To alleviate these shortcomings, cationic groups can be permanently incorporated into the backbone of chitosan by a process called quaternization. Quaternization permanently cationizes chitosan, which has been proven to improve its solubility, mucoadhesion, antimicrobial activity, permeability through biological membranes, and bioavailability [59].

The antimicrobial activity of quaternized chitosan is the result of the high number of positive charges on the surface of the polymer. The cationic groups bind strongly to the negatively charged phospholipids, proteins, and polysaccharides of the cell membranes of fungi and bacteria, causing membrane destabilization, intracellular‌ leakage, oxidative stress, and cell death. Quaternization also reduces the number of intermolecular hydrogen bonds, therefore increasing the flexibility of chitosan chains and its hydration properties [60]. Several synthetic methods for the quaternization of chitosan have been used, for example, N-trimethylation, imidazolium grafting, and quaternary salt coupling method being the most studied [61].

**Figure 1:**
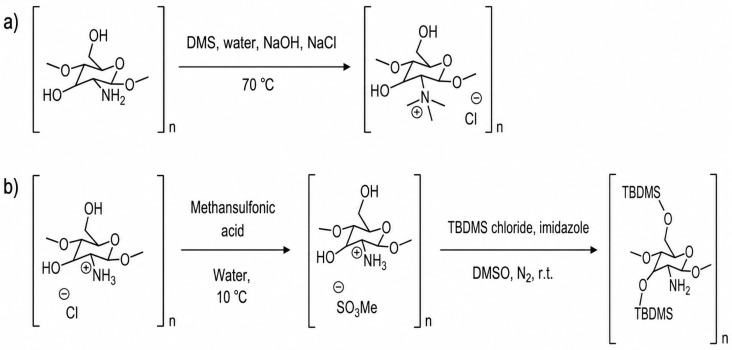
Schematic representation of the chemical modification of chitosan. (**a**) Synthesis of N,N,N-trimethyl chitosan (TMC) via quaternization of chitosan using dimethyl sulfate (DMS) under alkaline conditions. (**b**) Preparation of 2,3-di-O-TBDMS-protected chitosan through mesylation followed by selective hydroxyl protection using tert-butyldimethylsilyl chloride (TBDMS-Cl) and imidazole.

- *N*-Trimethylation:

The N-trimethylation of chitosan has been reported as one of the earliest and most common methods of quaternization, where methylation occurs on the amino groups present at the C-2 position on the chitosan molecule. This yields N,N,N-trimethyl chitosan (TMC), a permanently cationic derivative of chitosan with very good water solubility over a wide pH range [62]. Recent research has described TMC synthesis as being achieved through direct methylation of the amine moiety using methyl iodide, dimethyl sulfate, or methyl chloride under basic conditions, presumably via stepwise methylation of amino groups to give quaternary ammoniums. The degree of quaternization achievable has been shown to depend on reaction time, temperature, reagent concentration, and solvent composition [49]. Figure 1 highlights the schematic reaction of trimethylation.

The addition of trimethyl groups can have an important effect on the physicochemical properties of chitosan, namely through the increased solubility of chitosan in water. Native chitosan is insoluble in water above a pH of 6.5 due to deprotonation of amino groups and wide-ranging intermolecular hydrogen bonding. Trimethylation disrupts the formation of crystalline and hydrogen-bonded domains, and improves the solubility of chitosan over a wide range of pH [63].

Biologically, trimethyl chitosan has been found to have stronger antimicrobial and antifungal activity compared with native chitosan, because the increased cationic charge density allows stronger attraction between positively charged quaternary ammonium groups and negatively charged fungal cell membranes, which triggers cell membrane disruption and permeability. As several researchers have reported that TMC improves paracellular transport by reversibly opening epithelial tight junctions, it is a promising candidate for mucosal and transmucosal drug delivery systems. The antifungal effects of TMC depend on the degree of quaternization and substitution pattern: increased quaternization usually improves membrane interactions and fungicidal activity, although high substitution may accompany undesirable steric effects and reduced polymer–biological interactions. Therefore, the degree of substitution must be controlled and studied carefully [64,65].

Owing to its solubility in water, biocompatibility, and mucoadhesive properties, TMC has been studied widely in the fields of nanoparticles, ocular and nasal drug delivery, vaccine carriers, antifungal coatings, and controlled drug delivery. TMC has been demonstrated to display superior biological activity when compared to unmodified chitosan. O-quaternary ammonium salt chitosan derivatives developed by Wang et al. achieved a 100% inhibition rate against *Aspergillus* species and *Candida albicans* at 100 ppm with derivatives that possessed medium-length alkyl chains and high substitution degrees. In contrast, unmodified chitosan was only fungistatic, with no meaningful inhibitory action up to a concentration of 500 ppm. The results show that quaternization prompts a marked increase in fungicidal activity due to improved charge density and hydrophilic–hydrophobic balance [66].

Sajomsang et al. likewise synthesized water-soluble quaternized chitosan derivatives and examined their antifungal activity against Trichophyton rubrum, Trichophyton mentagrophytes, and Microsporum gypseum. These authors also observed that the degree of substitution and the type of quaternary ammonium substituents applied influenced the extent of these compounds’ inhibition. The derivatives were considerably more effective than native chitosan, which suggests that quaternization is an effective route for improving membrane-targeting antifungal activity [67]. In addition to its antimicrobial and antifungal effects, TMC is a promising drug delivery agent. The mucoadhesive affinity and transient tight junction opening ability of trimethyl chitosan have been widely investigated in developing drug delivery systems for the nasal, ocular, pulmonary, and oral routes of administration. The increased permeation could be used to improve the bioavailability of hydrophilic macromolecules such as peptides, proteins, and vaccines.

- Imidazolium Grafting:

Imidazolium grafting is a method of surface modification by which ionic imidazolium groups are chemically combined with the chitosan backbone. Imidazolium is a positively charged heterocyclic compound that occurs in ionic liquids. It has good antimicrobial, thermal, and electrochemical properties. Typically, the grafting mechanism occurs covalently: imidazole derivatives are grafted via alkylation, nucleophilic substitution or click chemistry reactions to the amino or hydroxy groups of chitosan. Chitosan is usually reacted with chlorinated or brominated imidazolium precursors in an alkaline medium. Under controlled basicity conditions, cationic graft copolymers of chitosan can be formed [68].

A major advantage of imidazolium-functionalized chitosan is its dual microbial action: (1) the positively charged imidazolium ring electrostatically adsorbs to the negatively charged microbial membrane; (2) hydrophobic groups on the imidazolium ring can penetrate and disrupt the lipid bilayer of the membrane. This generally results in strong antifungal and antibacterial effects even at relatively low concentrations [69]. Imidazole-grafted polycations also show increased antifungal activities. Wu et al. prepared five imidazole-functionalized chitosan derivatives, i.e., imidazole, 2-Methylimidazole, 4-Methylimidazole, 2-Ethylimidazole, and benzimidazole, and studied their antifungal activities against *Phytophthora nicotianae*, *Fusarium graminearum*, and *Rhizoctonia solani*. The benzimidazole chitosan derivative displayed antifungal activities towards *P. nicotianae* and *R. solani* at 42% and 50%, respectively, at a concentration of 0.5 mg/mL, while chitosan exhibited antifungal activity of 17% against *P. nicotianae*. The benzimidazole chitosan derivative showed a notably higher inhibition rate against *R. solani*, reaching up to 99% even at a starting concentration of 1.0 mg/mL, in contrast to the only 38% inhibition rate with unmodified chitosan, confirming the result of increased antifungal activities due to imidazolium grafting [70]. However, Ma and co-workers prepared a series of imidazole acid-functionalized chitosan derivatives that showed considerably improved antibacterial, antioxidant, and antitumor properties compared to native chitosan, mainly owing to the improved ionic interactions, electron transfer ability, and membrane permeability of the imidazolium-containing functionalities [71].

Imidazolium grafting greatly improves the physicochemical properties of chitosan. The high ionic nature of the imidazolium ring results in improved water dispersibility, ionic conductivity, thermal stability, and surface activity. In addition, the amphiphilic nature of several imidazolium derivatives makes them efficient at interacting with hydrophobic drugs and biological membranes, such that these systems may be useful as advanced drug delivery systems. Another property of imidazolium-functionalized chitosan is its production of ROS and its induction of oxidative stress within bacterial cells, which leads to an increase in membrane lipid, protein, and nucleic acid damage and improved fungicidal efficacy. Moreover, imidazolium-grafted derivatives also exhibit excellent antibiofilm activity, interfering with fungal adhesion and disrupting the organized structure of the biofilm. Multifunctional imidazolium-grafted chitosan derivatives are currently being explored for antifungal coatings, wound dressing, antimicrobial hydrogel scaffolds for tissue engineering and stimulus-responsive nanocarriers [71,72].

**Figure 2:**
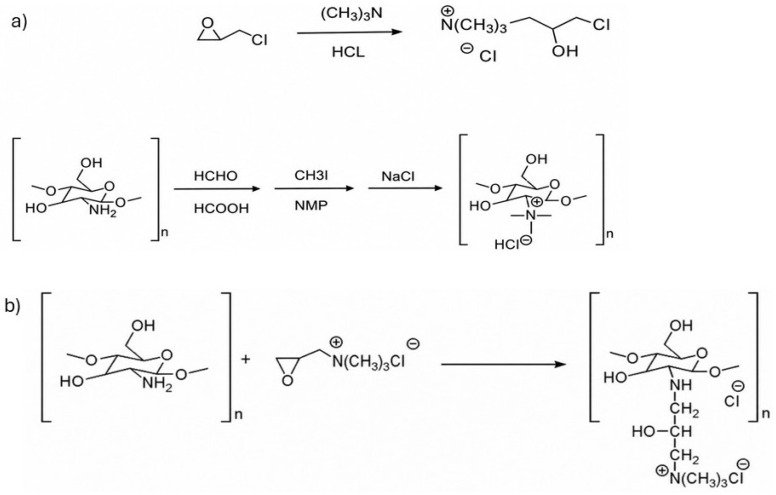
Synthetic approaches for the preparation of quaternized chitosan derivatives. (**a**) Two-step synthesis involving the preparation of glycidyl trimethylammonium chloride (GTMAC) from epichlorohydrin and trimethylamine (**top**), and the synthesis of TMC via methylation of chitosan using formaldehyde, formic acid, and methyl iodide (**bottom**). (**b**) Direct grafting of GTMAC onto chitosan through epoxide ring opening to produce HACC.

- Quaternary Salt Coupling Strategy:

Quaternary salt coupling is a form of indirect quaternization in which preformed quaternary ammonium groups are covalently coupled to chitosan. The inclusion of additional cationic groups with other structures in the chitosan polymer chain is accomplished without meaningful chain degradation by reaction of quaternary ammonium reagents such as glycidyl trimethylammonium chloride (GTMAC), 3-chloro-2-hydroxypropyl trimethylammonium chloride (CHPTAC) or brominated quaternary salts with amino or hydroxyl groups of chitosan through reactions known as nucleophilic substitution or epoxy ring opening. The products consist of permanently charged quaternary ammonium functionalities spaced throughout the polymer backbone. The synthetic pathways for the preparation of TMC and HACC are illustrated in Figure 2.

Quaternary salt coupling considerably increases the aqueous solubility and density of cations, because the ionic ammonium groups are highly hydrated, and the delivered charge can modulate swelling, mucoadhesion and the interaction with microbial membranes. Also related to film-forming ability and rheology is the ability to increase the hydrophilicity of the material. The earliest example of chitosan modified by GTMAC showed N-monoalkylation, improving water solubility and cationic character. The introduction of quaternary ammonium groups in place of hydroxyl groups in the polymer backbone broke intermolecular hydrogen bonding and increased hydration, which improved swelling and film-forming properties of the polymer. Liu et al. described soluble quaternary ammonium derivatives of chitosan prepared from CHPTAC with a DS of 0.3 to 0.5. These derivatives showed increased antibacterial activity and aqueous dispersibility compared to native chitosan, which were attributed to the positive charge from the quaternary ammonium moieties [49].

Antifungal mechanisms of quaternary salt-coupled chitosan derivatives have been associated with membrane disruption. The cationic polymer interacts electrostatically with negatively charged fungal cell-wall components like glucans, mannoproteins, and phospholipids, resulting in increased membrane permeability and the leakage of intracellular materials. Additionally, the hydrophobic substituents on some of these quaternary salts are known to ease membrane passage through lipid bilayers, and subsequently increase antifungal activity. In recent years, the use of click chemistry has expanded the possibilities of coupling. Tan et al. synthesized quaternized chitosan derivatives containing triazoles via azide–alkyne click reactions and revealed the synergistic effects of the triazoles and quaternary ammonium groups on increasing water solubility and antifungal activity by membrane interaction and inhibition of microbial proliferation [73]. One advantage of quaternary salt coupling is its ability to readily install multifunctional derivatives, including those designed for pharmacological, targeting, or diagnostic purposes. The rational selection of reagents enables the simultaneous modulation of antimicrobial activity, drug-loading capacity, biodegradability, and stimulus responsiveness to meet specific application requirements. Consequently, quaternary salt coupling is an ideal and viable way to generate next-generation chitosan-based antifungal biomaterials that are stable, wide-spectrum-antimicrobial, biocompatible, and drug-applicable [65,69,71,72].

### 2.2. Physicochemical Properties Governing Chitosan’s Antifungal Efficacy

Many physicochemical properties have been shown to influence the antifungal activity of chitosan and its derivatives. Molecular weight, degree of deacetylation, degree of substitution, surface charge density, solubility, crystallinity, hydrophilic–hydrophobic balance, and polymer conformation are important characteristics that influence the behavior of chitosan-based products when interacting with fungal cell membranes [74,75]. These features impact the permeability and uptake of membranes, bioadhesion, and overall fungicidal efficiency.

Chitosan is a polycationic linear polysaccharide. It is formed by β-(1→4)-bonded D-glucosamine and N-acetyl-D-glucosamine. The amino groups in the polysaccharide structure make them polycationic, since the amino groups are protonated in the acidic environment, forming positively charged sites on the polysaccharide chain. These protonated amino groups are primarily responsible for the interaction of chitosan with negatively charged fungal cell surfaces, including membrane phospholipids, glucans, mannoproteins, and intracellular components. One of the most important parameters influencing antifungal activity is DD. The DD determines the number of free amino groups available for protonation and electrostatic interaction. Chitosan is available in three degrees of deacetylation, i.e., low to medium (55–75%), and high (75–98%). Chitosan, with a higher DD, showed a larger number of positively charged amino groups, resulting in stronger attraction toward fungal membranes and greater membrane disruption. Several studies have confirmed the direct relationship between DD and antifungal activity. Jung et al. demonstrated that highly deacetylated chitosan (99%) exhibited significantly enhanced antibacterial activity due to increased amino group density and stronger electrostatic interactions with microbial membranes [76]. Similarly, Wang et al. reported that chitosan with approximately 83% deacetylation showed higher inhibitory action against several bacterial strains compared with lower-DD chitosan samples. Increased protonation capacity at higher DD values enhances fungal membrane permeability, leading to leakage of intracellular electrolytes, proteins, and nucleic acids [77]. However, a higher DD may reduce the flexibility of the polymeric chains and thus reduce mechanical properties.

Another factor involves molecular weight (MW), which influences polymer chain flexibility, diffusion behavior, membrane interaction, and the film-forming layer. Low-molecular-weight chitosan (less than 100 KDa) generally exhibits improved penetration into fungal cell walls and intracellular spaces, whereas high-molecular-weight chitosan (more than 700 KDa) tends to form polymeric films around microbial cells, restricting nutrient transportation and oxygen exchange deprivation. Qin et al. evaluated chitosan samples with different molecular weights and observed that antimicrobial activity varied depending on polymer size. Chitosan, with optimized molecular weight, demonstrated stronger inhibitory effects against *Escherichia coli* and *Staphylococcus aureus* [78]. Similarly, Liu et al. reported that molecular weight significantly affects antibacterial activity against *E. coli*, with intermediate-molecular-weight fractions showing significant biological activity due to higher diffusion into the membrane [79].

Another parameter is degree of substitution (DS), which determines the degree of quaternary ammonium group introduced into the chitosan backbone. The higher the DS, the more protonation there will be, which will increase the solubility; electrostatic interaction with the fungal cell membrane will thus increase the antifungal effect. Thus, an optimal DS is always appreciated as compared to maximum DS. Maximum DS may result in high cytotoxicity, reduced flexibility of the polymer, and reduced biodegradability.

Another critical physicochemical property is surface charge density, especially in quaternized derivatives of chitosan. The presence of quaternary ammonium moieties results in the formation of positive charges and hence an electrostatic interaction with the fungal cell membrane. Higher levels of cationicity contribute to better membrane disruption, osmotic instability, and internal leakage. Wang et al. prepared O-quaternary ammonium salt chitosan derivatives that completely inhibited *C. albicans* and *Aspergillus fungi* at a concentration of 100 ppm in cases where there were high substitution degrees and moderate alkyl chain lengths. Unlike chitosan derivatives, native chitosan had partially fungistatic properties, even at concentrations of 500 ppm [66]. These results indicate that cationic charge density and hydrophobic chain optimization are crucial factors governing antifungal potency.

However, the hydrophilic/hydrophobic ratio of the derivatives is also critical in determining the antifungal activity of chitosan derivatives. While hydrophilicity enhances water solubility and dispersion of the polymers, hydrophobicity is involved in permeation through the cell lipid bilayer membranes. There is no fixed ratio reported for chitosan derivatives; thus, an amphiphilic ratio is always preferred to yield the maximum antifungal effect. Moderate levels of hydrophobicity provide the most effective fungicide properties. According to Sajomsang et al., the antifungal activity of the quaternized chitosan derivatives against *Trichophyton rubrum*, *Trichophyton mentagrophytes*, and *Microsporum gypseum* depended greatly upon the hydrophilic/hydrophobic ratio and level of substitution of the polymer [67].

Solubility is another crucial parameter that plays an important role in the biological behavior of the compound. Natural chitosan can be dissolved in the acid medium (typically at pH < 6.5) because protons are required to protonate the amino groups (-NH_2_ to -NH_3_^+^; pKa ≈ 6.3–6.5) to break the hydrogen bonds. However, limited solubility under physiological conditions limits further medical applications. Quaternization and carboxymethylation significantly increase solubility in water by adding charges or hydrophilic groups to the backbone structure of the polymer. Tan et al. produced a quaternized derivative of chitosan that contains triazole units and revealed increased water solubility with increased antifungal activity [73]. The study lacked quantitative measurements of solubility; based on turbidity, the researchers measured the extent of solubility across the entire tested pH range, including neutral and alkaline conditions. However, the crystal structure and hydrogen bonding ability of chitosan can also determine its capacity to prevent the growth of microorganisms and fungi. Chitosan is derived from chitin, which can be found in three forms—alpha, beta, and gamma crystal structures—each distinguished by a unique pattern and interactions between the molecules. The first form is marked by a highly organized pattern of antiparallel chains and high hydrogen bonding ability [80]. On the other hand, β-chitin forms fewer intermolecular interactions and is more flexible, which could enhance its membrane interactions and biological activities. There is no specific value mentioned in the literature that specifies the range of percentage crystallinity of chitosan. Various chemical treatments, such as quaternization, alkylation, and graft copolymerization, lower the degree of crystallinity by breaking intermolecular hydrogen bonds. This increases the chain’s flexibility, due to which functional groups will be more exposed to having membrane interactions with fungal cells, thus possibly having a substantial antifungal effect [29,81].

Moreover, physicochemical characteristics affect the ability of chitosan derivatives to act as antibiofilm agents against fungi. Positively charged derivatives may interfere with adhesive events in fungi and destabilize extracellular polymeric substances (EPSs) present within biofilms. Chitosan derivatives containing imidazolium groups and quaternization possess significant antibiofilm activity due to their ability to increase membrane permeation and produce ROS [72]. In conclusion, we can say that the above-mentioned parameters are interconnected and dependent on each other. Higher DD of the polymer will result in enhanced protonation and zeta potential, due to which the membrane interaction of the polymer will be high, resulting in improved antifungal activity. Similarly, a high degree of quaternization means a higher degree of substitution, and solubility will be greatly enhanced, which lowers crystallinity, improves membrane penetration, and enhances the antifungal effect. Rational optimization of these physicochemical properties through targeted chemical modification is therefore essential for designing highly efficient chitosan-based antifungal biomaterials and drug delivery systems.

## 3. Advances in Quaternary Ammonium Chitosan-Based Formulations for Antifungal Applications

The combination of several unique properties makes QAC useful not only as an antifungal but also as a potential drug delivery system. These include inherent antifungal properties, the presence of permanent positive charges on the surface, the ability to adhere to mucous membranes, and high synthetic flexibility. The drawback of natural chitosan, which limits its application, is associated with the dependence of its antifungal effect on protonation of its amino groups. As a result, such an agent is effective only in an acidic medium and completely inactive at pH > 6.5, when its solubility and biological activity cease. Quaternization overcomes this drawback since it endows the compound with permanent and non-pH-dependent positive charges, thereby making it soluble at any pH value and strengthening its polycationicity, responsible for its antifungal activity. The mechanism of the latter is related to the electrostatic binding of the positively charged compounds to the negatively charged elements of the cell membrane, resulting in a breach of the protective cell barrier, followed by an osmotic imbalance and loss of intracellular contents. The following subsections describe the ways in which this chemistry has been utilized and developed from the structure–activity optimization of the polymer backbone to its incorporation within nanocomposites, biofilm-targeting molecules, and delivery systems (Table 3) [82].

### 3.1. Direct Antifungal Activity of QA-Chitosan Derivatives

#### 3.1.1. Structure–Activity Relationships: Charge Density and Electron-Withdrawing Groups

**Figure 3:**
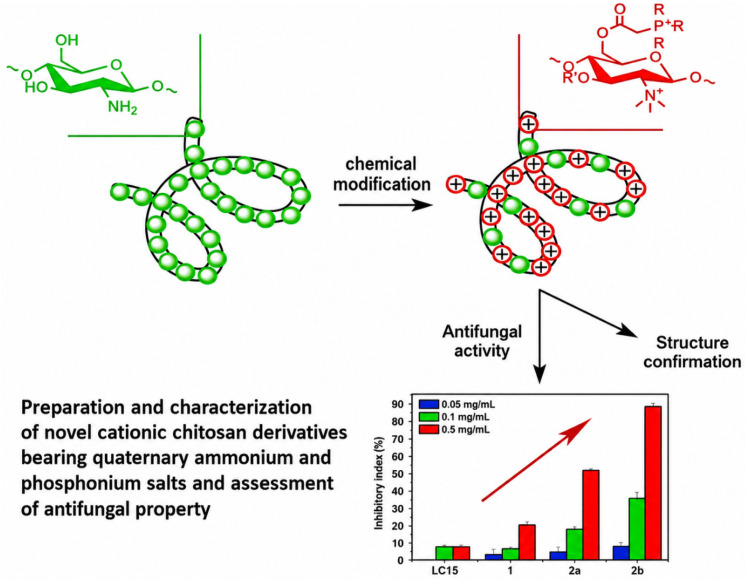
Schematic illustration depicting the chemical synthesis of quaternary ammonium and phosphonium-functionalized chitosan derivatives, structural analysis, and assessment of antifungal potential. Modification of chitosan through chemical means resulted in cationic derivatives that exhibited antifungal performance, especially the phosphonium derivative [83].

Early studies in this field revealed an unambiguous relationship between activity against fungi and structural modification, where increased antifungal activity was correlated with greater cationic charge density and electron-withdrawing properties of the substituted group in chitosan. Specifically, Tan et al. developed QA and phosphonium chitosan derivatives by sequentially carrying out trimethylation, chloroacetylation, and quaternization on chitosan, making it more soluble in water and thermally stable, as depicted in Figure 3. Among several species of phytopathogenic fungi, such as *Phomopsis asparagi*, *Fusarium oxysporum*, *Colletotrichum lagenarium*, and *Watermelon fusarium*, the triphenylphosphonium-substituted derivative showed maximum antifungal activity by providing an almost 80% inhibition rate at 0.5 mg/mL due to its greater cationic charge density and electron-withdrawing property. This showed that, apart from quaternization, the targeted functionalization of the substituent on the polymer is important for improving the antifungal property of the substance [83]. The study was an early-stage in vitro study. However, the study should have also included a detailed mechanistic explanation of how the phosphonium group can improve the antifungal efficacy of chitosan. Furthermore, for the optimization of polymer design, a robust SAR could be mentioned, highlighting the different spacer lengths, alkyl and aromatic phosphonium derivatives, their degree of substitution, and phosphonium density.

**Figure 4:**
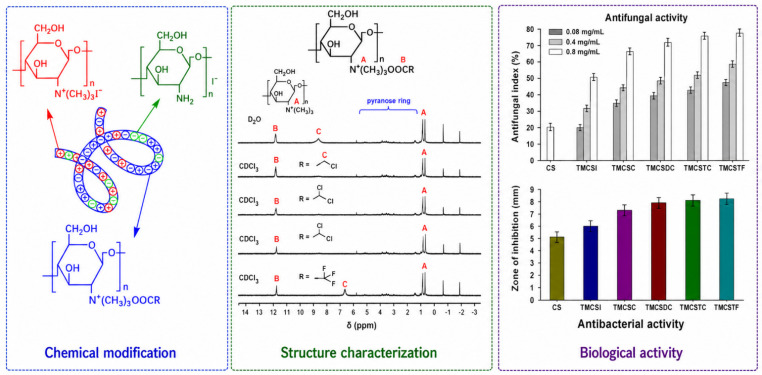
Schematic depicting the preparation of quaternary ammonium salts bearing halogenated acetate groups (TMCSTF, TMCSTC, TMCSDC, and TMCSC). Structural conformation of the synthesized derivatives was determined by ^13^C nuclear magnetic resonance spectroscopy, which showed characteristic resonances corresponding to the QA and halogenated acetate moieties. The antifungal and antimicrobial activities demonstrated that the halogenated acetate-functionalized derivatives exhibited enhanced performance compared with chitosan and TMCSI [32].

Further support for the role of electron-withdrawing substituents was established using halogen functionalization. To solve the problem associated with low fungicidal properties and poor solubility of native chitosan, a series of derivatives of trimethyl chitosan QA salts with halogenated acetate moieties was prepared and tested on agriculturally significant fungi. In this case, the QA moiety imparted a permanent positive charge, whereas the halogen-substituted groups improved electron-withdrawing capacity and interaction with the cell membranes. The antifungal efficacy correlated with the magnitude of the electron-withdrawing ability of the halogen, that is, trifluoroacetate (TMCSTF) > trichloroacetate (TMCSTC) > dichloroacetate (TMCSDC) > chloroacetate (TMCSC), as shown in Figure 4. The most effective compound inhibited the growth of *F. Oxysporum*, *Botrytis cinerea*, and *P. asparagi* with efficiency up to 77.15% at a concentration of 0.8 mg/mL. From the mechanism study, it was found that the compound had a twofold effect, namely, adsorption through electrostatic interactions and destabilization of the permeability and leakage of cell proteins, thus killing the fungal cells [32]. This investigation synthesizes four halogenated acetates. An elaborate SAR must be included to highlight the significance of modification, i.e., degree of quaternization and deacetylation, polymer chain length, charge density, and hydrophobicity. Chemical stability studies of the developed polymers should also have been done to ensure their pH, thermal, and enzymatic storage conditions.

#### 3.1.2. Restoring Water Solubility While Preserving Biocompatibility

Another approach involves the enhancement of chitosan’s poor solubility in physiological and alkaline solutions, along with the incorporation of additional biological activities. Imidazolium quaternary ammonium derivatives of chitosan with 6-O substitution, synthesized by chloroacetylation, represent a good example of such modification. The presence of permanent cations prevented the formation of hydrogen bonds responsible for the lack of water solubility, thus obtaining fully water-soluble compounds. Against the pathogens *B. cinerea* and *Gibberella zeae*, derivatives with longer alkyl chains, like 2-(N,N,N-trimethyl)-6-O-(1-butylimidazole) acetyl chitosan chloride (NBMC), and primary amines like 2-(N,N,N-trimethyl)-6-O-(2-aminobenzimidazole) acetyl chitosan chloride (2NPhMC) displayed over 85% inhibition at a concentration of 1.0 mg/mL, which was a marked improvement from the inhibition rate shown by chitosan. This effect could be due to the strong electrostatic interactions causing blockage of nutrient transport, as well as damage to the cell envelope of fungi. The derivatives were also characterized by antioxidant properties and had no cytotoxic effects on HaCaT keratinocytes, proving that increased solubility does not affect their biocompatibility [84]. However, the study lacks quantitative SAR, which is needed to develop rational design of more potent derivatives. The stability of developed derivatives could also have been investigated, along with resistance development studies for their long-term effectiveness. For studying the efficacy of the polymer in persistent infections, biofilm inhibition studies could be conducted. More relevant cell lines could have been used to establish the toxicity of the polymer.

**Figure 5:**
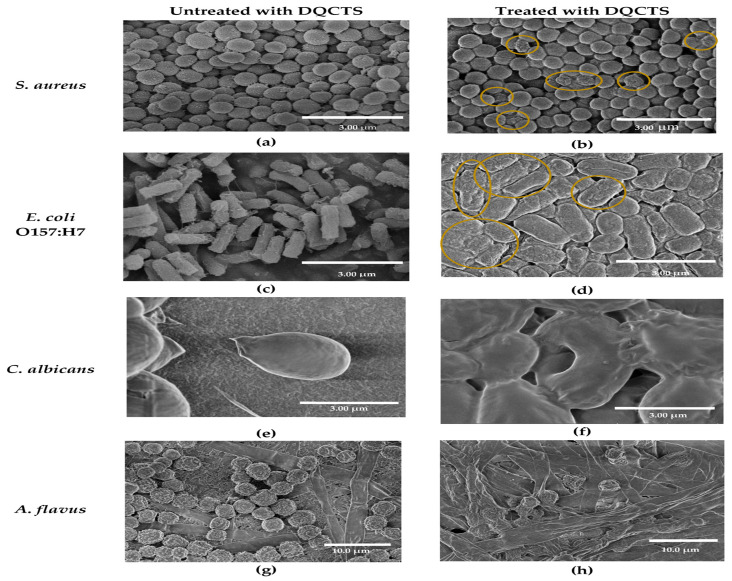
SEM photomicrographs show the morphological changes in microorganisms before and after treatment with DQCTS. (**a**,**b**) *S. aureus* cell displaying cell shrinkage and surface deformation. (**c**,**d**) *E. coli* O157:H7 showing membrane disruption, wrinkling, and cracking. (**e**,**f**) *C. albicans* exhibiting collapsed structures and extensive surface damage with pore formation. (**g**,**h**) *A. flavus* spores and hyphae showing disintegration and dissolution of fungal structures. Yellow circles indicate representative damaged regions. Scale bars: 3 μm (*S. aureus*, *E. coli* O157:H7, *C. albicans*) and 10 μm (*A. flavus*) [85].

The interplay between deacetylation, quaternization, and antifungal properties has been analyzed in depth in the work of Kim et al., where a highly deacetylated quaternized chitosan (DQCTS) was synthesized by subjecting the chitosan to deacetylation to almost 100%, followed by quaternization using 3-chloro-2-hydroxypropyltrimethylammonium chloride. Since the level of quaternization depends on the number of free amino groups, deacetylation allowed for achievement of an extremely high quaternization (99.15%) degree in comparison with a traditionally modified chitosan (69.71%). The derivative had a very high cationic charge density along with good water solubility. In biological tests, the substance showed high antifungal properties, especially towards *C. albicans*, whose minimum inhibitory concentration (MIC) and minimum fungicidal concentration (MFC) were found to be equal to 15.6 µg/mL and 31.3 µg/mL, respectively, much lower than the analogous indicators for *S. aureus* and *E. coli* (250 µg/mL). DQCTS inhibited the development of *Aspergillus flavus* in two days. Damage to fungal cells could be observed by scanning electron microscopy (SEM) as perforations in *C. albicans* cells and destruction of *A. flavus* spores and mycelia, as depicted in Figure 5. Additionally, qRT-PCR results revealed inhibition of genes associated with virulence, which include genes involved in the production of aflatoxins, namely, AflaO and AflaR. Notably, the DQCTS retained relatively low cytotoxicity towards HaCaT cells, indicating that maximizing the charge density by way of deacetylation and quaternization greatly improves the antifungal activity [85]. This work could be significantly enhanced by optimizing the degree of quaternization and molecular weight to enhance efficacy and reduce toxicity. The polymer can be evaluated against drug-resistant fungal strains and biofilm eradication assessment. Long-term stability and safety studies could be included to ascertain the shelf-life of the polymer.

- Escalating charge density through multiple quaternization

Based on the principle of charge density, several research works aimed to maximize antifungal activity by incorporating multiple QA groups within every repeating unit of chitosan. Aminopyridine-based compounds with triple quaternary ammonium clusters (TQCSP1 and TQCSP2) showed significant antifungal potential compared to the native chitosan and commercial oligosaccharins towards *Phytophthora capsici*, *Rhizoctonia solani*, *F. oxysporum*, and *Fusarium solani*. Specifically, TQCSP1 showed a maximum inhibition index of 91.94% towards *P. capsici* at a concentration of 0.8 mg/mL. Incorporation of triple-cationic clusters considerably boosted the charge density and solubility in water, resulting in higher interaction between the compounds and the cell wall/membrane of the fungi. This destroyed the cell membrane, causing osmotic imbalance and loss of cellular contents [49]. Only two pyridine derivatives were developed, so a robust SAR is missing in the study, which could have included the position of pyridine substitution, spacer length, number of QA groups, molecular weight, and alkyl chain length. A mechanistic pathway like apoptosis or membrane disruption, or multiple ways to establish the ways in which the polymer is acting as an antifungal, could be added to the study. Stability studies can be included to ensure the storage conditions of the derivatives. The same findings have been obtained when testing other analogous compounds, which were pyridine-based triple-quaternized derivatives of chitosan, prepared via grafting epoxypropyltrimethylammonium chloride to doubly quaternized compounds. These derivatives had high antifungal activity towards *Watermelon fusarium*, *F. oxysporum*, and *P. asparagi*, with derivative 8 demonstrating high inhibition, especially against watermelon *Fusarium* (1.0 mg/mL). Importantly, the biocompatibility of these derivatives towards 3T3-L1 cells remained relatively high [86]. Only three isomers of pyridinium were created. A systematic SAR could have been conducted to understand the effect of position of substitution, charge density, quaternization degree, and molecular weight. This research reports both antifungal and cell toxicity studies, but selectivity towards fungal cells as compared to mammalian cells is not discussed. Chemical stability studies need to be established. Overall, all of these findings prove the correlation between increasing positive charge concentration and high antifungal activity through enhanced electrostatic interaction with the fungal membrane, without compromising mammalian cell compatibility.

- Metal coordination and nanoparticle hybrids

Another unique research direction utilizes the metal-chelating capability of chitosan along with the antifungal properties of metal ions. Il’ina et al. managed to synthesize quaternized N-(propyl) derivatives of water-soluble chitosan with degrees of substitution from 40 to 58%, using glycidyltrimethylammonium chloride, and then obtained a Cu(II)–chitosan complex of a 53%-substituted derivative with 1.3% copper content. The effectiveness of these complexes was tested against various yeast-like fungi, such as *Saccharomyces cerevisiae*, *Rhodotorula rubra*, and *C. albicans*, as well as filamentous fungi such as *F. oxysporum*, *Alternaria alternata*, and *Cladosporium herbarum*. Of all the test subjects, the Cu–chitosan complex demonstrated noticeable activity against *F. oxysporum*, which is one of the most common and economically damaging fungal plant pathogens, reaching efficient activity at concentrations of 250–500 µg/mL. It is evident that coordination of metal ions with an inherent antifungal potential further enhances the biologically active nature of the cationic polymer [87]. The study reports antifungal studies but lacks the mechanistic evidence for how metal–chitosan complexes kill fungal strains. The researchers have developed 40–58%-substituted derivatives, but a systematic evaluation is really needed to identify the optimal substitution degree. Toxicity studies should be included along with stability studies. Metal augmentation is exemplified at its highest level in nanoparticle-based methods. Quaternary trimethyl chitosan–silver nanoparticles (QTMC-AgNPs) were fabricated using a green approach of one-pot reduction via compressed hydrogen gas, leading to the formation of quasi-spherical nanoparticles of an average size of around 12.5 nm. Such an approach has also solved another problem related to the poor solubility of chitosan at neutral and alkaline pH. Although QTMC had no significant antimicrobial effect, the nanocomposite formed between QTMC and AgNPs was able to display potent antifungal and antibacterial properties. It inhibited Sclerotium rolfsii completely and *F. oxysporum* by 76.67%. Furthermore, the nanocomposite created zones of inhibition of 30 mm and 26 mm against *E. coli* and *S. aureus*, respectively. The quaternized polymer serves as a solubilizing matrix, stabilizing agent, and charged carrier for improving the antimicrobial properties of the AgNPs [88].

### 3.2. Antibiofilm Strategies and Advanced Therapeutic Platforms Based on QA-Chitosan

Controlling biofilms is a significant difficulty in the fight against infectious illnesses since biofilm-dwelling microbes can be up to 1000 times more resistant to disinfection than free-floating ones. In addition to bacterial biofilms, fungal biofilms have recently been a fast-growing issue for diseases associated with devices. Even more challenging to treat, these infections worsen patients’ quality of life, increase mortality and mobility, lengthen hospital stays, and raise medical expenses [37]. These structured microbial communities, which are encased in a self-produced EPS matrix, exhibit remarkable resilience, withstanding traditional antimicrobial treatments and adapting to and recovering from environmental and therapeutic stresses, thereby requiring the development of innovative antibiofilm strategies [89].

The major drawback associated with traditional antimicrobials is poor residence of the molecule on biofilms, which usually leads to ineffective killing of the microbes. Jung et al. designed an amphiphilic quaternary ammonium-modified chitosan polymer (CS612) through an acid-free method of modification. In vitro testing with MIC and MBC assays on Gram-positive bacteria and fungi revealed effective antimicrobial activity against the tested organisms. The presence of the amphiphilic character resulted in the increased ability of CS612 to attach itself to the surface of biofilms, ensuring its localization within the target area and efficient antimicrobial action. Furthermore, CS612 displayed good biocompatibility towards mammalian cells, indicating its use for topical antifungal treatment and wound care [90].

An analogous multifunctional method was reported based on montmorillonite (MMT) nanosheets containing 5-fluorocytosine (5-FC), copper ions, and QAC. This QAC/MMT/5-FC-Cu nanocomposite showed wide-spectrum antimicrobial properties against Gram-positive bacteria (*S. aureus*), Gram-negative bacteria (*E. coli*), and fungal pathogens (*C. albicans*). The improved efficacy of this antimicrobial material was associated with the coordinated binding of 5-FC to copper ions, as well as the QAC layer. Release profile tests showed a rapid burst release effect that was capable of significantly lowering the microbial burden, as well as subsequent sustained release providing an extended period of action. In addition, studies on wound healing in vivo demonstrated efficient infection management, improved tissue regeneration, and biocompatibility of the proposed system. This study demonstrated the great prospect of QAC/MMT/5-FCCu nanocomposite as an effective means for therapy of complicated wounds with bacterial and fungal infections [91].

To overcome the poor biofilm penetration and limited skin permeability associated with conventional antifungal treatments, a dissolvable microneedle (MN) system incorporating terbinafine (TB) and QAC was developed. The TB/QAC MNs demonstrated a synergistic antifungal effect against Trichophyton mentagrophytes, significantly enhancing therapeutic performance. The microneedles effectively disrupted fungal biofilms and degraded EPSs, improving drug access to the infection site. In a murine dermatophytosis model, TB/QAC MNs accelerated fungal eradication, reduced treatment duration, and promoted skin healing compared with terbinafine alone. These findings highlight the potential of TB/QAC MNs as an effective strategy for treating superficial fungal infections [92].

Functional group engineering has also been employed to improve the bioactivity of quaternized chitosan derivatives. Schiff-base-containing glyoxylate derivatives were prepared for trimethyl chitosan iodide (TMCI) and HACC by ion-exchange modification. These derivatives possessed strong antifungal activity towards Gloeosporium cingulatum, with a maximum inhibition percentage of over 84.88% at 1.0 mg/mL. Among the Schiff bases of the two derivatives, aminopyridine-modified TMCI Schiff bases presented higher antifungal activity, demonstrating the importance of incorporating Schiff bases in derivatives. Moreover, these Schiff-base derivatives demonstrated excellent antioxidant activity, as their scavenging capacity of DPPH free radicals exceeded 92.40%. However, their biocompatibility remained satisfactory, with their cell viability over 80.14% at 1000 µg/mL [93].

Electrospun fibers containing QA-chitosan are an emerging format with a dual purpose, such as as wound dressings and as local antifungal delivery matrices, especially in chronic wound infections with fungal superinfection. Malik et al. developed TMC-capped gold–silver bimetallic nanoparticles (Au-Ag-TMC-NPs) and incorporated them into polyvinyl alcohol/chitosan nanofibers via electrospinning, producing a nanofibrous wound dressing with fiber diameters of 100–350 nm, a nanoparticle size of 108.3 ± 8.4 nm, and a zeta potential of +54.4 ± 1.8 mV. The system had strong broad-spectrum antimicrobial activity with MIC ranging between 0.390 and 3.125 μg/mL^−1^ and strong inhibition of *C. albicans* and multidrug-resistant (MDR) bacterial strains of *S. aureus*, *E. coli*, *P. aeruginosa*, and *C. albicans* mediated by membrane disruption, DNA condensation, and efflux pump inhibition. In vivo testing in MDR-infected wound models showed rapid wound healing, with 99.34% of the wounds closed within 12 days, which was supported by an increase in the expression of the endogenous antimicrobial biomarker chloramphenicol and photoacoustic imaging of the wound [94]. However, to bring the developed formulation to clinical settings, long-term safety and biodistribution studies are required. It is also suggested to compare the developed formulation with existing marketed wound care products. Collectively, these platform-level studies show that the same cationic, charge-driven chemistry of QA-chitosan is responsible for direct fungicidal action, which can be harnessed within nanocomposites, microneedles, and nanofibers to confront biofilm-protected and device-associated fungal infections that resist conventional therapy.

**Table 3:** Research advancements in QA-chitosan derivatives and their antifungal potential.

QA-Chitosan Derivative	Key Modifications and Physicochemical Characteristics	Target Microorganisms	Key Outcomes and Antimicrobial Mechanisms	Reference
QA and phosphonium-functionalized chitosan (e.g., triphenylphosphonium–chitosan)	Sequential trimethylation, chloroacetylation and quaternization; cationic QA and phosphonium salts; improved water solubility and thermal stability.	*P. asparagi*, *F. oxysporum*, *C. lagenarium*, and watermelon *Fusarium*	Triphenylphosphonium derivative most potent (80% inhibition at 0.5 mg/mL). Higher cationic charge density and the strong electron-withdrawing phosphonium center enhance electrostatic binding to the fungal envelope.	[83]
Halogenated trimethyl chitosan quaternary ammonium salts (TMCSC, TMCSDC, TMCSTC, TMCSTF)	Trimethylation followed by quaternization with halogenated acetates; electronegative halogens raise membrane-disrupting hydrophobicity; water-soluble.	*F. Oxysporum*, *B. cinerea*, and *P. asparagi*	Antifungal index up to 77.15% at 0.8 mg/mL; potency rises with halogen electron-withdrawing strength (TMCSTF > TMCSTC > TMCSDC > TMCSC). Electrostatic cell-wall binding increased the outer-membrane permeability, which led to leakage of essential proteins.	[32]
Imidazolium-based 6-O-quaternary ammonium chitosan (NBMC, 2NPhMC)	Chloroacetylated intermediate; permanent cationic charge disrupts intramolecular H-bonding → full water solubility; intrinsic antioxidant activity.	*Botrytis cinerea* and *Gibberella zeae*	Inhibitory index >85% at 1.0 mg/mL (NBMC, 2NPhMC). Polycationic “electrostatic walls” block nutrient exchange and damage the cell envelope; non-toxic to HaCaT keratinocytes.	[84]
DQCTS	~100% deacetylation followed by quaternization (CHPTAC); degree of quaternization 99.15% (vs. 69.71% for conventional QCTS); highly charge-dense and water-soluble.	*C. albicans* and *A. flavus*	*C. albicans* MIC 15.6/MFC 31.3 µg/mL; *A. flavus* growth arrested by day 2. SEM showed large holes in *C. albicans* and dissolved *A. flavus* spores/mycelia; downregulation of virulence and aflatoxin genes (AflaO, AflaR).	[85]
Aminopyridine chitosan with triple quaternary ammonium clusters (TQCSP1, TQCSP2)	Triple-cationic clusters markedly raise positive charge density; broad water solubility; enhanced cell-wall binding affinity.	*P. capsici*, *R. solani*, *F. oxysporum*, and *F. solani*	TQCSP1 peak inhibitory index 91.94% against *P. capsici* at 0.8 mg/mL; outperformed native chitosan and commercial oligosaccharins. Strong polycationic interaction disrupts barrier permeability that led to osmotic imbalance and leakage of cellular constituents.	[49]
Pyridinium-based triple-quaternized chitosan (compounds 7, 8, 9)	Epoxypropyltrimethylammonium chloride grafted onto doubly quaternized intermediates; triple QA salts increase charge density; water-soluble at neutral/alkaline pH.	Watermelon *Fusarium*, *F. oxysporum*, and *Phomopsis asparagi*	High antifungal indices; compound 8 strongly inhibited watermelon *Fusarium* at 1.0 mg/mL. Strong electrostatic disruption of the fungal cell wall; low toxicity and high biocompatibility in 3T3-L1 cells.	[86]
Quaternized N-(propyl) chitosan–Cu(II) metal complex	Low-MW chitosan quaternized with glycidyltrimethylammonium chloride (40–58% degree of substitution); Cu(II) complex of the 53%-substituted derivative (1.3% copper) by dialysis; confirmed by IR/^1^H NMR, Cu by atomic emission spectrometry.	Yeast-like: *Saccharomyces cerevisiae*, *Rhodotorula rubra*, and *Candida albicans*; mycelial: *F. oxysporum*, *Alternaria alternata*, and *Cladosporium herbarum*	The Cu(II) complex is effective against *F. oxysporum* (one of the ten most common fungal plant pathogens) at 250–500 µg/mL. Coordinated metal ions augment the antifungal action of the cationic polymer backbone.	[87]
QTMC-AgNPs	Single-step green reduction using compressed H_2_ as a clean reducing agent; quasi-spherical NPs 12.5 nm; water-soluble polymer acts as matrix and capping agent.	*Sclerotium rolfsii* and *F. oxysporum* (also *E. coli*, *S. aureus*)	Plain QTMC inactive; nanocomposite gave 100% inhibition of *S. rolfsii* and 76.67% of *F. oxysporum*; inhibition zones 30 mm (*E. coli*) and 26 mm (*S. aureus*). Polymer solubilizes, stabilizes, and potentiates the silver payload (bimodal action).	[88]
Amphiphilic quaternary ammonium chitosan (CS612)	External acid-free synthesis; amphiphilic structure designed for biofilm anchoring; cytocompatible.	Gram-positive bacteria and fungi (planktonic and biofilm)	Favorable MIC/MBC; latches onto bacterial and fungal biofilms and kills resident organisms in situ, giving efficient localized action with good mammalian cytocompatibility.	[90]
QCS/MMT/5-FCCu montmorillonite nanocomposite	MMT nanosheets co-loaded with 5-FC, Cu ions, and a QAC coating; in situ 5-FC-Cu coordination; biphasic (burst and sustained) release.	*Staphylococcus aureus*, *Escherichia coli*, and *Candida albicans*	Broad-spectrum activity from 5-FC-Cu coordination plus the QAC coating. Burst release rapidly lowers microbial load; sustained release prolongs action. In vivo: effective infection control, accelerated tissue repair, and minimal toxicity.	[91]
TB/QAC MNs	Dissolvable microneedle array co-loading TB and QAC; physically breaches the skin barrier for intradermal delivery.	*Trichophyton mentagrophytes* (dermatophyte)	Synergistic antifungal effect; disrupts fungal biofilm and degrades EPSs to improve drug access. Murine dermatophytosis: faster fungal eradication, shorter treatment, and improved skin healing vs. terbinafine alone.	[92]
Schiff-base-functionalized TMCI and HACC	Glyoxylate-bearing Schiff bases introduced into TMCI and HACC by ion exchange; antioxidant.	*Gloeosporium cingulatum*	>84% inhibition at 1.0 mg/mL; aminopyridine-based TMCI Schiff bases are most active. Strong antioxidant activity; cell viability >80% at 1000 µg/mL.	[93]
Electrospun Au-Ag-TMC bimetallic nanoparticle/PVA–chitosan nanofibers	TMC-capped gold–silver bimetallic NPs (108.3 ± 8.4 nm; zeta potential +54.4 ± 1.8 mV) embedded in PVA/chitosan nanofibers by electrospinning; theranostic wound dressing.	*C. albicans* and MDR bacterial strains	MIC 0.390–3.125 µg/mL; 99.34% wound closure within 12 days. Acts via membrane disruption, DNA condensation, and efflux pump inhibition; upregulation of an endogenous antimicrobial peptide; tracked by photoacoustic imaging.	[94]

## 4. Safety and Toxicity Considerations of QA-Chitosan Systems

Generally, QA-chitosan derivatives have a good biocompatibility profile, but the degree of quaternization, charge density, molecular weight, and type of substituent play a significant role in their safety profile. Based on SAR, unless there is a counter-ion effect, increased quaternization increases the water solubility and antifungal activity by increasing the amount of permanent positive charge density, which may also result in increased binding to mammalian cell membranes, with a potential increase in cytotoxicity. Molecular weight also impacts the cellular uptake, tissue penetration, and biological distribution, and other hydrophobic/aromatic substitutions may influence antifungal effects and biocompatibility. There are very few studies that have reported toxicity assessments of QAC. Wu et al. formulated quaternary ammonium chitosan/rhamnolipid composite nanoparticles and conducted acute in vivo toxicity studies using a zebrafish model and compared it with sodium hypochlorite. The developed formulation showed lower toxicity than sodium hypochlorite. The study lacks comprehensive mammalian biocompatibility studies, including cytotoxicity, hemocompatibility, and long-term toxicity studies, which are required for clinical translation. The study did not include the mechanism responsible for lesser toxicity [95]. Similarly, Andreica et al. developed quaternary ammonium chitosan-based hydrogels as biocidal products and assessed their toxicity and biocompatibility both ex vivo on red blood cells (RBCs) of Swiss mice and in vivo on white Swiss adult male mice. On RBCs, significant hemolysis was not reported. Systemic and local toxicity studies also reported no toxic effect in mice. The study lacks long-term safety and pharmacokinetic studies on cell lines, which could have added value to the developed product for its clinical translation [96]. Another group, Cui et al., developed four chitosan derivatives by using 2-thiophenecarboxaldehyde, 2-furancarboxaldehyde, 2-pyridinecarboxaldehyde and benzaldehyde. Thereafter, they conducted a toxicity assessment on L929 mouse fibroblast cells in a range of 1-to-1000-microgram concentration and reported significant compatibility with no toxic effect. This shows that alteration in native chitosan does not negatively impact its biocompatibility [97].

**Figure 6:**
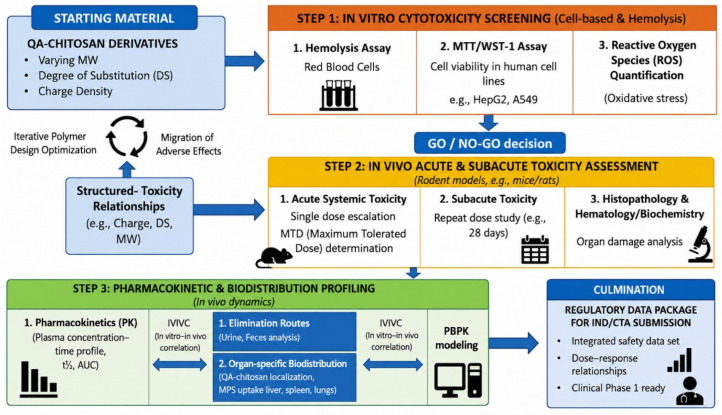
Schematic representation of the safety and toxicity evaluation framework for QA-chitosan-based antifungal formulations.

Importantly, many safety assumptions are based on native chitosan, which cannot be straightforwardly transposed in all chitosan derivatives obtained by quaternization due to the changes in physicochemical and biological properties suffered by the polymer. Figure 6 depicts the schematic structure of the safety and toxicity assessment framework of the QA-chitosan-based antifungal formulations, which illustrates the sequential assessment of physicochemical characterization, in vitro cytotoxicity and genotoxicity screening, in vivo acute and subacute toxicity studies, pharmacokinetic and biodistribution profiling, and regulatory risk evaluation needed to translate the formulations into clinical practice [98,99].

## 5. Challenges with QAC Clinical Translation

Despite promising physicochemical properties with respect to antifungal effects, as evidenced in vitro and in vivo, there are major hurdles encountered during QAC synthesis, including controlling the rate of reaction and keeping the native chitosan in a solubilized form (by maintaining acidic pH), so that the amino groups become freely available for reaction [36,100]. In addition, there may be a possibility of side reactions such as O-quaternization, over-substitution, or unwanted crosslinking, which can degrade the quality of synthesized chitosan [36,100,101]. Furthermore, these reactions include harsh reaction conditions that can diminish the molecular weight, impacting viscosity, formation of film, and therapeutic activity [100,102]. There are challenges in the purification of synthesized QAC, especially removal of residual solvents and reagents, as QAC becomes highly water-soluble [103]. Still, several factors affect clinical translation, such as batch-to-batch variability during the synthesis of modified chitosan, which may result in differences in molecular weight, degree of substitution, and deacetylation [36,100,102]. There is a need for reproducible, economic, and scalable synthesis processes that can adhere to GMP requirements. In addition, an exhaustive qualitative and quantitative assessment with respect to biodegradation, immunogenicity, long-term toxicity, and pharmacokinetics should be strictly established, specifically when making formulations for systemic administration.

## 6. Future Perspectives and Opportunities

The growing interest in QAC derivatives for antifungal drug delivery has opened several promising avenues for future research and clinical application. Owing to their excellent biocompatibility, biodegradability, enhanced solubility, and intrinsic antimicrobial activity, QAC-based systems are increasingly being explored as advanced multifunctional platforms capable of addressing the limitations associated with conventional antifungal therapies. To take full advantage of these, future advancements need to revolve around developing targeted therapies, exploring wound-healing applications, making intelligent drug delivery systems, and overcoming drug resistance [38].

Historically, the development of biopolymers like QAC derivatives was done primarily based on trial-and-error, where different parameters were adjusted blindly to achieve the desired result, but now things have changed vastly [91]. Nowadays, artificial intelligence, machine learning, and computational modeling have become an integral part of developing a rational framework for these derivatives, which helps in altering the biological and physiochemical profiles in situ before wet-lab synthesis.

### 6.1. Multi-Scale Molecular Modeling

The performance of QAC derivatives is influenced by various structural modifications that can be comprehended by the different simulations deployed across various spatial and temporal scales by computational workflows, which are mentioned below [104]:

- Quantum Chemical and Density Functional Theory (DFT) Calculations: Within the sub-nanometer scale, scientists employ DFT calculations to gain insights into the electronic properties and chemical stability of the specific quaternary ammonium (QA) head groups that are grafted onto the glucosamine backbone of the chitosan molecule. Using DFT, scientists can map the electrostatic potential surfaces of these molecules to gain a better understanding of their reactive landscape at the active sites of their molecules. This allows for the modeling of the quaternization reactions that occur at the QA head groups, examining aspects like the nucleophilic substitution kinetics of the Menshutkin reaction [105,106].
- All-Atom Molecular Dynamics (MD) Simulations: These simulations model the explicitly solvated behavior of QCS chains at the nanometer scale. They work by identifying the fluctuations in structural configurations of modified chitosan when coming into the vicinity of water molecules and counter-ions. These simulations provide insight into how the positively charged QA groups interact with the negatively charged lipid bilayers or cell-wall components of fungal pathogens, which is a critical component for antifungal applications. Furthermore, these simulations highlight the mechanics of how the alkyl tails of QCS insert into and disrupt the fungal cell membranes [106].
- Coarse-Grained (CG) Modeling: Because full-scale polymer chains and macroscopic phenomena—such as self-assembly into antifungal nanoparticles, micellation, or hydrogel gelation—exceed the temporal constraints of all-atom simulations, coarse-grained models are adopted. By grouping clusters of atoms into single “beads,” CG simulations allow the exploration of larger structural dynamics, illustrating how polymer–fungicide composites aggregate, encapsulate cargo, or interact over extended interfaces [106].

### 6.2. Machine Learning and Predictive Frameworks

While physics-based molecular simulations map specific localized interactions, AI and data-driven algorithms accelerate the high-throughput screening of massive virtual libraries of QAC derivatives. Quantitative structure–activity relationship (QSAR) models use machine learning classifiers (such as Random Forest, Support Vector Machines, or Deep Neural Networks) to screen candidate polymers based on calculated molecular features. In high-performance antimicrobial polymer design, ML pipelines process critical parameters, including the calculated partition coefficient, total hydrophobic vs. hydrophilic balance, degree of polymerization, and total cationic charge density, to predict the minimum inhibitory concentration against prominent fungal pathogens like *Candida albicans* [44]. In addition to predicting chemical properties, generative AI models can suggest chemical variations, like determining the optimal length of the N-alkyl chains (usually between C13 and C15) for maximizing membrane disruption while minimizing red blood cell lysis or suggesting green synthetic pathways for achieving high substitution without degrading the polymer [77].

### 6.3. Roadmap for Preclinical to Clinical Translation

This review shows that multiple preclinical studies have demonstrated QAC effectiveness, but scientists must go through a complex process to bring this discovery from laboratory testing to human clinical studies. Research needs to be done to link macromolecular chemistry with regulatory science and translational medicine. The QAC derivatives show strong antifungal effects against many different fungi in laboratory tests, yet the development of a thorough validation system, which will transform these initial findings into authorized medical treatments for people, has yet to be done.

The first roadblock is the scale-up phase, moving from developing milligram-scale in a laboratory to kilogram-scale Current Good Manufacturing Practice (cGMP) production. Since chitosan is a natural biopolymer sourced from chitin (crustaceans or fungi), it has inherent variability in molecular weight distribution and baseline degree of deacetylation (DD) [104]. To ensure reproducibility from batch to batch, strict control is required over the chemical functionalization of the polymer—including controlling the degree of quaternization (DQ) and alkyl chain grafting. Standardized analytical techniques such as proton nuclear magnetic resonance spectroscopy and Gel Permeation Chromatography should be used to measure these parameters. Additionally, the polymer must be able to withstand industrial sterilization techniques such as autoclaving, gamma irradiation, or ethylene oxide treatment without undergoing degradation of any kind [105].

Apart from the challenges in manufacturing these QCS derivatives, there also exists comprehensive toxicological and functional validation in advanced in vitro and in vivo models, which they need to pass before being able to be used on humans. The main toxicological concern for polymers with a high positive charge is their potential to interact with the negatively charged membranes of mammalian cells and cause lysis. Therefore, any potential QCS derivative must demonstrate that it does not cause hemolysis of human red blood cells and that it is not cytotoxic to human cells of the target tissue at concentrations far more than its minimum inhibitory concentration [106].

Additionally, the in vivo pharmacokinetics and biodistribution of the polymer must be monitored to ensure that there is no toxic accumulation in major organs such as the liver and kidneys. Furthermore, the polymer’s ability to eradicate persistent pathogens in vivo must be demonstrated in appropriate animal models of vulvovaginal candidiasis, invasive aspergillosis, or cutaneous biofilms.

The last stage of translating the use of these derivatives from preclinical to clinical will involve the creation of human clinical trials and obtaining regulatory approvals, such as Investigational New Drug applications with global regulatory bodies like the US Food and Drug Administration. The initial use of these derivatives clinically should be confined to indications with clear unmet medical needs and low-risk delivery routes. Topical and local applications of antifungal hydrogels for diabetic foot ulcer infections, functionalized wound dressings, or ophthalmic drops for fungal keratitis have a more straightforward regulatory pathway compared to intravenous administration, since their systemic biodistribution and toxicity risks are very limited [44]. Human trials for this new antifungal agent must systematically progress through the various clinical trial phases. Initially, Phase I clinical trials will be conducted with healthy volunteers. These trials will focus strictly on the safety and local tolerability of the agent. Following these initial trials, Phase II clinical trials will be conducted to evaluate the appropriate dosing of the antifungal agent. Finally, large-scale Phase III clinical trials will be conducted to evaluate the clinical efficacy of the agent against emerging multidrug-resistant strains.

## 7. Conclusions

QAC-based drug delivery systems have shown a very promising avenue for treatment against invasive, superficial, and multidrug-resistant fungal infections. The main drawback of native chitosan is its poor solubility in physiological conditions, which chemical quaternization overcomes to produce a biopolymer with permanent cationic character and exceptional biomedical versatility. These next-generation nanomaterials have a distinct dual-function modality that holds real therapeutic promise. The high-density positive charges of the polymer backbone produce a powerful antifungal action by targeting the negatively charged components of fungal cell walls and membranes. The QAC-based nanoparticles also function as drug delivery vehicles for antifungal drugs, improving their localized delivery, sustained release, and stability. Poorly aqueous-soluble antifungal drugs need additional strategies for their nanoparticulate formulation, such as QAC-coated nanocarriers, polymer–lipid hybrid NPs, and self-assembled amphiphilic QCh NPs. This process increases the efficacy of the treatment while reducing the systemic toxicity of the drugs. To utilize the full potential of QAC-based drug delivery platforms, innovations in multiple scientific and engineering fields are necessary to translate these systems from the laboratory to the clinic.

## Figures and Tables

**Table 1:** Overview of recent review articles related to QA-chitosan, including their major focus areas and research coverage.

S. No.	Year	Review Covers	Reference
1	2025	This review describes the structural features, mechanism of action, use in antimicrobial materials, drug and gene delivery, and tissue engineering applications of QA-based salts.	[43]
2	2020	This article describes the preparation and applications of QA derivatives of chitosan for drug delivery. The review summarizes the performance of these derivatives based on their cytotoxicity, biocompatibility, and ability to enhance drug absorption.	[33]
3	2024	This article describes the biomedical applications of chitosan and its derivatives, highlighting their roles in antibacterial therapy, cancer treatment, wound healing, and tissue engineering. It also emphasizes their use in targeted drug delivery, tissue regeneration, and infection control.	[44]
4	2024	This article describes the properties, preparation, and wide-ranging applications of a quaternized chitosan derivative. It highlights how quaternization overcomes the limitations of native chitosan by improving water solubility, biocompatibility, and biological activity.	[45]
5	2021	The study compares different modification strategies and reports that chitosan impregnated with quaternary ammonium salts exhibited the strongest antibacterial and antifungal activity. It also highlights the potential of other functionalized chitosan derivatives as promising platforms for controlled delivery of biologically active molecules.	[46]

**Table 2:** Functionalization strategies of chitosan and their effect on physicochemical properties with applications.

Modification Strategy	Reaction	Key Structural Changes	Effect on Physicochemical Properties	Functional Advantages	Major Applications	References
Schiff-Base Modification	Reaction of chitosan amino groups with aldehydes or ketones to form imine (–C=N–) linkages under mild conditions	Introduction of aromatic or aliphatic substituents through Schiff-base formation	Increased hydrophobicity, enhanced surface activity, improved stability, and altered membrane interactions	Improved antimicrobial, antifungal, antioxidant, and metal-chelating activities; serves as an intermediate for further derivatization	Antimicrobial, antifungal, antioxidant, metal-ion chelator, and synthesis of N-substituted chitosan derivatives	[50,51]
Carboxymethylation	Introduction of carboxymethyl groups using monochloroacetic acid under alkaline conditions	Formation of N-carboxymethyl, O-carboxymethyl, or N, O-carboxymethyl derivatives	Enhanced hydrophilicity, improved water solubility over a wide pH range, increased moisture retention, and film-forming	Excellent biocompatibility, improved swelling behavior, and functional versatility	Wound dressing, hydrogels, tissue engineering scaffolds, drug delivery systems, and biomedical coatings	[52,53]
Alkylation	Introduction of alkyl groups via reactions with alkyl halides, epoxides, or related compounds, predominantly at amino groups	Formation of N-alkylated or O-alkylated derivatives with hydrophobic or hydrophilic side chains	Modified hydrophilic–hydrophobic balance, improved membrane permeability, enhanced solubility, and polymer flexibility	Enhanced antimicrobial and antifungal activity through stronger membrane interactions; improved physicochemical stability	Controlled drug delivery, nanoparticles, gene delivery, antifungal formulations, and biomedical carriers	[54,55,56]
Acylation	Introduction of acyl groups using acid chlorides, acid anhydrides, or other acylating agents	Formation of N-acylated and/or O-acylated derivatives	Reduced crystallinity, improved solubility, enhanced thermal stability, mechanical strength, and tunable hydrophobicity	Improved drug-loading capacity, biocompatibility, sustained-release characteristics, and antimicrobial performance	Sustained-release drug delivery systems, antimicrobial coatings, biodegradable biomaterials, and tissue engineering applications	[57,58]

## Data Availability

No new data were created or analyzed in this study. Data sharing is not applicable to this article.

## Abbreviations

The following abbreviations are used in this manuscript:

2NPhMC	2-Aminophenyl Imidazolium-Modified Chitosan
5-FC	5-Fluorocytosine
*A. flavus*	*Aspergillus flavus*
*A. fumigatus*	*Aspergillus fumigatus*
AgNPs	Silver Nanoparticles
AI	Artificial Intelligence
Au-Ag-TMC-NPs	Gold–Silver Trimethyl Chitosan-Capped Bimetallic Nanoparticles
*B. cinerea*	*Botrytis cinerea*
*C. albicans*	*Candida albicans*
*C. auris*	*Candida auris*
cGMP	Current Good Manufacturing Practice
CG	Coarse-Grained
CHPTAC	3-Chloro-2-Hydroxypropyltrimethylammonium Chloride
COVID-19	Coronavirus Disease 2019
CS	Chitosan
DD	Degree of Deacetylation
DFT	Density Functional Theory
DNA	Deoxyribonucleic Acid
DQ	Degree of Quaternization
DQCTS	Highly Deacetylated Quaternized Chitosan
DS	Degree of Substitution
*E. coli*	*Escherichia coli*
EPS	Extracellular Polymeric Substance
FDA	Food and Drug Administration
*F. oxysporum*	*Fusarium oxysporum*
GTMAC	Glycidyl Trimethylammonium Chloride
HACC	Hydroxypropyltrimethyl Ammonium Chloride Chitosan
HIV/AIDS	Human Immunodeficiency Virus/Acquired Immunodeficiency Syndrome
IFIs	Invasive Fungal Infections
IR	Infrared Spectroscopy
MBC	Minimum Bactericidal Concentration
MDR	Multidrug-Resistant
MD	Molecular Dynamics
MFC	Minimum Fungicidal Concentration
MIC	Minimum Inhibitory Concentration
MMT	Montmorillonite
ML	Machine Learning
MN	Microneedle
MW	Molecular Weight
NBMC	N-Butyl Imidazolium-Mo
